# Adult Medulloblastoma Associated with Syringomyelia: A Case Report

**DOI:** 10.3969/j.issn.2095-3941.2012.02.011

**Published:** 2012-06

**Authors:** Ching-Chun Wang

**Affiliations:** Nepean Hospital, Kingswood NSW 2747, Australia

**Keywords:** syringomyelia, medulloblastoma, hydrocephalus, bradycardia

## Abstract

The association between cerebellar medulloblastoma and syringomyelia is uncommon and only found in pediatric patients. To date, adult medulloblastoma associated with syringomyelia has not been reported in the literature. Paroxysmal bradycardia is an uncommon clinical manifestation in posterior fossa tumors and likely to be vagally mediated via brainstem preganglionic cardiac motor neurons. This report introduces the diagnosis and treatment of a case of adult medulloblastoma associated with syringomyelia, which presented with paroxysmal bradycardia.

## Introduction

Medulloblastoma is an embryonal neuroepithelial tumor. It is the most common primary brain tumor in children, representing 25% of all pediatric brain tumors and around 30% to 40% of primary posterior fossa tumors ^[^^1^^]^. However, medulloblastoma only accounts for approximately 1% of adult primary brain tumors, the majority of which are diagnosed in the third and fourth decades of life ^[^^2^^]^. The tumor commonly arises from the roof of the fourth ventricle, which leads to a local invasion and distant metastases through the cerebrospinal fluid (CSF) ^[^^3^^]^. Obstructive hydrocephalus and syringomyelia can potentially develop due to the disturbance of the CSF flow at the craniospinal level. However, there have only been isolated pediatric cases of medulloblastoma with or without hindbrain herniation in association with syringomyelia, and there are no reports on adult cases in the literatures. The clinical symptoms and signs of medulloblastoma patients are mainly headache and disturbance of cerebellar functions. The manifestation of reflex bradycardia of vagal origin associated with posterior fossa tumors, presumably via mechanical and/or ischemic stimulation of preganglionic cardiac motor neurons in the external formation of the nucleus ambiguous and dorsal motor nucleus of the vagus nerves, is an uncommon clinical finding ^[^^4^^]^.

This report describes a 41-year-old man who had classic medulloblastoma and an extensive syrinx in association with cerebellar medulloblastoma, and later developed paroxysmal bradycardia shortly after his hospital admission.

## Case Report

A 41-year-old man was presented to the emergency department with progressively worsening suboccipital headache, ataxia, and diplopia. The patient had headache for 4 weeks, and it gradually increased in severity and persistence during this period. No clear precipitating and alleviating factors were identified. He also noticed that his walking gait became more unsteady and he tended to lean toward his left side. Diplopia had also been noticed a week before this presentation. No speech and language deficits, personality change, and difficulty in performing complex tasks were apparent. The patient was otherwise healthy without significant past medical or surgical histories.

On examination, the patient was alert and oriented to the place, time, and persons. He was in moderate distress due to his severe suboccipital headache. His body temperature was 37°C, blood pressure was 140/80 mmHg, heart rate ranged from 60 beats to 70 beats per minute, and his respiratory rate was 18 breaths per minute. Cranial nerve examination showed diplopia at all gazes and a left horizontal nystagmus. Cerebellar disturbance was found in the left upper and lower limbs, including dysdiadochokinesia and dysmetria in a finger-to-nose test and a positive heel–shin test. A wide-based standing posture and walking gait as well as a positive tandem walking test were also noticed. Other examinations were unremarkable.

**Figure 1:**
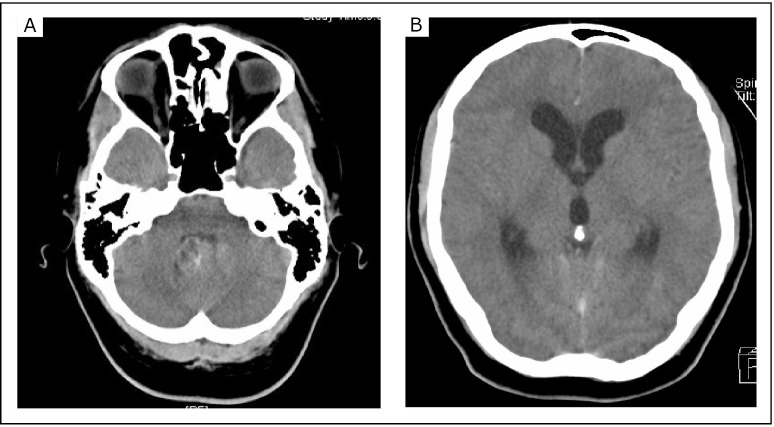
Contrast-enhanced CT revealing a lesion in the fourth ventricle (A), leading to obstructive hydrocephalus with a dilatation of the third and lateral ventricle (B).

A computed tomography (CT) scan of the head using an intravenous contrast revealed a solid lesion of 2.5 cm in diameter with lobulated cysts and mild contrast enhancement in the fourth ventricle (**Figure 1**). A nearly complete obstruction of the foramen magnum by cerebellar tonsillar herniation and a dilatation of the third and lateral ventricles with subependymal edema were also demonstrated (**Figure 1**).

The patient was then transferred to the intensive care unit (ICU), treated with dexamethasone, and then prepared for an urgent magnetic resonance imaging (MRI) study and neurosurgical intervention. Two hours after the ICU admission, paroxysmal sinus bradycardia was found and patient heart rate oscillated between approximately 30 beats and 70 beats per minute. The occurrence of transient bradycardia made it difficult to capture blood pressure using a non-invasive blood pressure monitor. The patient complained about intensified headache during the bradycardia period.

**Figure 2:**
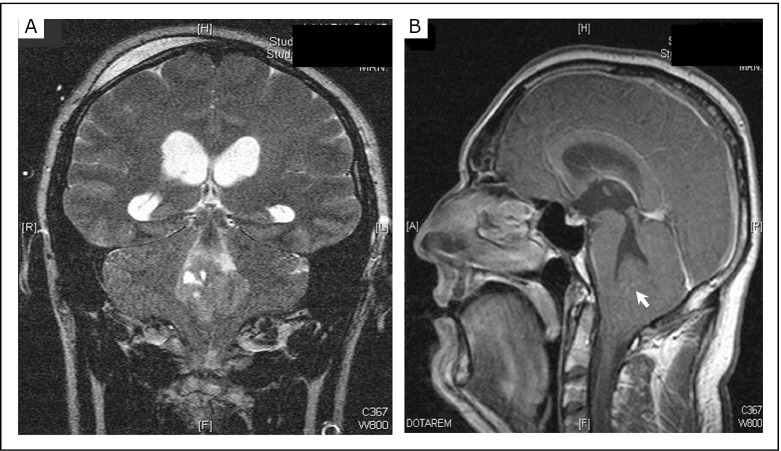
Coronal T2-weighted MRI (A) demonstrating a lesion in the fourth ventricle resulting in an outflow obstruction of the CSF at the foramina of Luschka and Magendie, and a dilatation of the third and fourth ventricles and the temporal horns of the lateral ventricles. Sagittal T1-weighted image with gadolinium enhancement (B) revealing the lesion with minimal contrast enhancement leading to a cranial displacement of the inferior medullary velum (arrow) and a cerebellar tonsillar herniation with nearly total obstruction of foramen magnum.

**Figure 3:**
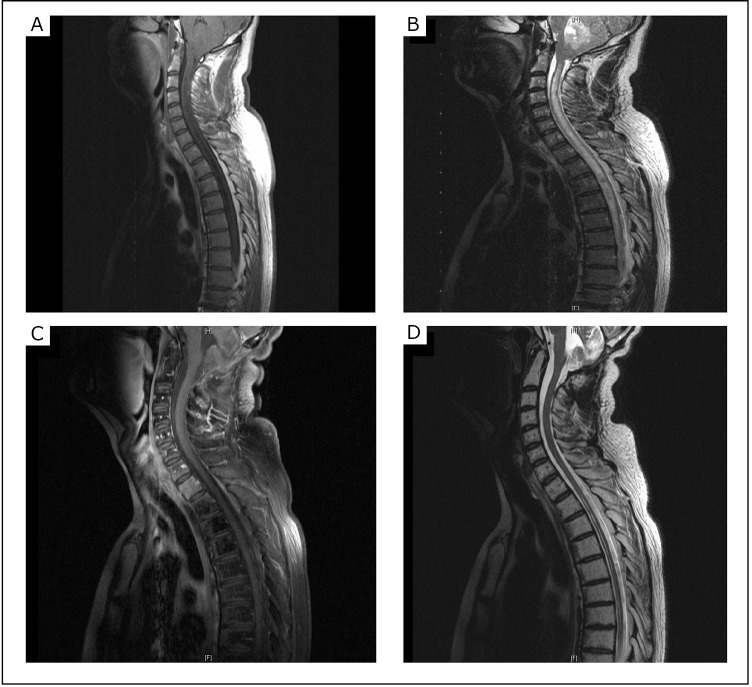
Pre-operative sagittal T1-weighted image with gadolinium enhancement (A) and T2-weighted image (B) showing syringomyelic cavity and no spinal, leptomeningeal spread of tumor. Post-operative sagittal T1-weighted image (C) and T2-weighted image (D) showing the regression of the syrinx.

He was then transferred to an operating theater for the insertion of an external ventricular drain. Intracranial pressure was 32 cm of water, and CSF was intermittently drained to stabilize this pressure. Later, an MRI of the brain confirmed the CT findings of hydrocephalus and cerebellar tonsillar herniation (**Figure 2**). The extension of the lesion to the left foramen of Luschka was further demonstrated. An MRI of the spine revealed a syrinx extending from the C2 to T9 vertebrae and no leptomeningeal seeding (**Figure 3**). The tumor was subsequently removed via suboccipital approach, and histopathological studies demonstrated classic medulloblastoma.

The patient’s presenting symptoms were resolved subsequently, and no postoperative focal neurological deficits were found. He was discharged a week later after undergoing outpatient radiation therapy, which involved a complete dose of craniospinal radiation with a boost on the primary tumor site. Two years after treatment, repeated magnetic resonance images showed the resolution of the syrinx and no clear radiological evidence of disease relapse (**Figure 3**).

## Discussion

Medulloblastoma is a primitive neuroepithelial tumor that arises from the external granular layer of the cerebellum and is considered as a Grade IV malignancy by the World Health Organization ^[^^5^^]^. Metastasis, young age, and residual tumor volume after resection have long been considered as poor prognostic factors in the pediatric population ^[^^6^^]^. Although similar factors have been studied in adults, the results of the studies are inconclusive ^[^^1^^]^. The main treatments of the lesion in adults are surgical resection and complete craniospinal radiotherapy along with a boost of radiation to the tumor bed. This treatment regimen yields an approximately 50% to 60% 5-year survival rate ^[^^1^^]^. Despite the common use of chemotherapy in pediatric patients, the role of adjunct chemotherapy in adults is still under investigation because of the different neoplastic biology, the rarity of the tumor in adults, and a paucity of prospective studies.

The pathogenesis of syringomyelia remains unclear since Charles Estienne first described it in 1546 ^[^^7^^]^. Although posterior fossa lesions with or without cerebellar tonsillar herniation have long been postulated as a potential cause of syringomyelia, this association is not commonly observed. Syringomyelia related to medulloblastoma has only been described in isolated pediatric cases (**Table 1**) ^[^^5^^, ^^8^^–^^10^^]^. Many hypotheses on its pathogenesis have been proposed and the details of these hypotheses have been discussed previously^[^^11^^]^. In this case, syringomyelia is likely associated with the obstruction of CSF outflow at the foramen magnum level. This association is supported by the spontaneous regression of the syrinx after the tumor resection.

**Table 1:** Syringomyelia cases in association with medulloblastoma.

	Hamlat et al. ^[^^5^^]^	Hinokuma et al. ^[^^8^^]^	Klekamp et al. ^[^^9^^]^	Tachibana et al. ^[^^10^^]^	Present case
Ages (years)/Gender	8/M	16/F	8/F	5/F	42/M
Clinical symptoms	Headache, vomiting, gait instability, and head tremor	NR	Headache, meningism, cerebellar ataxia, dysarthria, and vomiting	NR	Suboccipital headache, unstable gait with a wide base, nystagmus and diplopia
Symptoms secondary to syringomyelia	No	No	No	No	No
Site of a syrinx	T6-T8	L2-S1	C2-T8	C2-C7	C2-T9
Presence of cerebellar herniation	Yes	No	Yes	Yes	Yes
Presence of hydrocephalus	Yes	No	Yes	NR	Yes
Presence of autonomic reflexes	No	NR	No	No	Yes
Postoperative regression of a syrinx	Yes	Yes	Yes	Yes	Yes

NR: not recorded

Paroxysmal bradycardia associated with posterior fossa tumors is also an uncommon clinical finding. It is likely to be of vagal origin via ischemic and/or mechanical stimulation of preganglionic cardiac motor neurons in the external formation of the nucleus ambiguus and dorsal motor nucleus of the vagus nerves ^[^^4^^]^.

Although adult medulloblastoma is an uncommon primary brain tumor, it should be a differential diagnosis of posterior fossa lesions. The association of the tumor with syringomyelia should also be considered in patient management. Clinicians must appreciate the important autonomic reflexes requiring a prompt clinical response.
